# Enhancing root physiology for increased yield in water-saving and drought-resistance rice with optimal irrigation and nitrogen

**DOI:** 10.3389/fpls.2024.1370297

**Published:** 2024-05-08

**Authors:** Danping Hou, Kun Liu, Shikun Liu, Juncai Li, Jinsong Tan, Qingyu Bi, Anning Zhang, Xinqiao Yu, Junguo Bi, Lijun Luo

**Affiliations:** ^1^ Shanghai Agrobiological Gene Center, Shanghai, China; ^2^ Institute for Agri-food Standards and Testing Technology, Shanghai Academy of Agricultural Sciences, Shanghai, China; ^3^ Agronomy College, Jilin Agricultural University, Changchun, China

**Keywords:** water-saving and drought-resistance rice, irrigation regime, nitrogen fertilizer application rate, grain yield, root morphology and physiology

## Abstract

**Objectives:**

Water-saving and drought-resistance rice (WDR) plays a vital role in the sustainable development of agriculture. Nevertheless, the impacts and processes of water and nitrogen on grain yield in WDR remain unclear.

**Methods:**

In this study, Hanyou 73 (WDR) and Hyou 518 (rice) were used as materials. Three kinds of nitrogen fertilizer application rate (NFAR) were set in the pot experiment, including no NFAR (nitrogen as urea applied at 0 g/pot), medium NFAR (nitrogen as urea applied at 15.6 g/pot), and high NFAR (nitrogen as urea applied at 31.2 g/pot). Two irrigation regimes, continuous flooding cultivation and water stress, were set under each NFAR. The relationships between root and shoot morphophysiology and grain yield in WDR were explored.

**Results:**

The results demonstrated the following: 1) under the same irrigation regime, the grain yield of two varieties increased with the increase of NFAR. Under the same NFAR, the reduction of irrigation amount significantly reduced the grain yield in Hyou 518 (7.1%–15.1%) but had no substantial influence on the grain yield in Hanyou 73. 2) Under the same irrigation regime, increasing the NFAR could improve the root morphophysiology (root dry weight, root oxidation activity, root bleeding rate, root total absorbing surface area, root active absorbing surface area, and zeatin + zeatin riboside contents in roots) and aboveground physiological indexes (leaf photosynthetic rate, non-structural carbohydrate accumulation in stems, and nitrate reductase activity in leaves) in two varieties. Under the same NFAR, increasing the irrigation amount could significantly increase the above indexes in Hyou 518 (except root dry weight) but has little effect on Hanyou 73. 3) Analysis of correlations revealed that the grain yield of Hyou 518 and Hanyou 73 was basically positively correlated with aboveground physiology and root morphophysiology, respectively.

**Conclusion:**

The grain yield could be maintained by water stress under medium NFAR in WDR. The improvement of root morphophysiology is a major factor for high yield under the irrigation regime and NFAR treatments in WDR.

## 1 Introduction

With the continuous innovation of high-yield rice cultivation technology in China, rice yield has increased year by year. However, it is generally based on the cultivation and management mode with high input in water and nitrogen (Yan et al., 2022). A key problem with rice cultivation in China is the insufficient utilization of water and nitrogen resources. How to improve the efficiency of resource utilization has become a hot research topic (Song et al., 2018). The root is the primary organ for absorbing nutrients and water in rice. It is also a necessary place for the synthesis of various ions, organic acids, hormones, and amino acids. The formation of yield and aboveground growth have a close relationship with root morphology and physiology (Rebouillat et al., 2009). They are complicated by the regulation of water and nitrogen. Previous studies have found that an appropriate increase in nitrogen fertilizer application rate (NFAR) can increase the root length, root surface area, and root volume in rice; enhance the absorption of nutrients by plants; and ultimately increase rice yield (Wu et al., 2016). However, high NFAR will inhibit the root oxidation ability and zeatin (Z) + zeatin riboside (ZR) contents in roots, reducing rice yield (Devrim et al., 2017). Irrigation regime is also a considerable cultivation measure affecting rice yield (Belder et al., 2004). Appropriate water stress may not significantly reduce rice yield (Kumar et al., 2019). Although severe water stress did not inhibit root growth, it significantly reduced rice yield (Thakur et al., 2018).

Water-saving and drought-resistance rice (WDR) not only has the characteristics of high yield and excellent quality in rice but also has the traits of water-saving and drought resistance in upland rice (Luo et al., 2019). It has a strong ability to resist drought (Xu et al., 2015b). Previous research has demonstrated that unreasonable irrigation regimes and NFAR will inhibit root growth and development, thus affecting the formation of grain yield and quality in rice (Liu et al., 2018). High NFAR under water stress could significantly increase root dry weight, root volume, root length, and grain yield (Huang et al., 2019). Nonetheless, it was also observed that the root activity and the yield of grains were the highest under water stress coupled with medium NFAR (Xu et al., 2018). However, the effects and mechanisms of irrigation regime and NFAR on root morphology, physiology, and grain yield in WDR are not clear.

The objectives of this study were to investigate the differences in grain yield and root morphophysiology between paddy rice and WDR under the three kinds of NFAR [no nitrogen applied (0N), medium nitrogen applied (MN; 15.6 g/pot), and high nitrogen applied (HN; 31.2 g/pot)] and two irrigation regimes (continuous flooding cultivation and water stress) set for each NFAR. Root dry weight, root oxidation activity, root bleeding rate, root total absorbing surface area, root active absorbing surface area, Z + ZR contents in roots, leaf photosynthetic rate, non-structural carbohydrate (NSC) accumulation in stems, and nitrate reductase (NR) activity in leaves were measured, which strongly correlated with grain yield. This research will give recommendations for fertilizer and water management in WDR cultivation, as well as sustainable development in agriculture, and provide deeper comprehension of the mechanism of high yield under the condition of water and nitrogen interaction in WDR.

## 2 Materials and methods

### 2.1 Experimental station

The pot experiment was carried out at the Zhuanghang Experimental Station, Shanghai Academy of Agricultural Sciences, Shanghai, China (30°88′89″N, 121°38′51″E) from 2019 to 2020. The experiment soil was a mixture of sand and soil, and the weight of sand and soil was 1:2, containing 17.5–18.6 g/kg organic matter, 20.2–21.6 mg/kg ammonia nitrogen, 4.53–4.64 mg/kg nitrate nitrogen, 39.2–40.6 mg/kg available phosphorus, and 128.3–130.0 mg/kg available potassium.

### 2.2 Experimental design

Hanyou 73 (WDR) and Hyou 518 (paddy rice, control variety) were used as materials. They had similar growth times and were both three-line hybrid *indica* varieties. Seedlings were cultivated over the course of each year in a seedbed with a sowing date of May 26–27. On June 29 and 30, six hills, each containing one seedling, were transplanted. Six treatments in this study involved water and nitrogen factors. Three nitrogen (as urea) were applied, including no nitrogen applied (0N), medium nitrogen applied (MN; 15.6 g/pot), and high nitrogen applied (HN; 31.2 g/pot). All nitrogen fertilizer treatments were applied at once as basal fertilizer. Two irrigation regimes (continuous flooding cultivation and water stress) were included under each NFAR. For the continuous flooding cultivation regime, a 2–3-cm water depth was maintained with continuous flooding. For the water stress regime, the irrigation amount was 60% of the continuous flooding cultivation regime. A laboratory pump (KPHM100, Kamoer, Shanghai, China) was equipped to ensure that the flow rate of the hose was consistent during watering. The watering time of water stress treatment was 60% of the time of continuous flooding cultivation.

Before transplanting, as a base fertilizer, 30 g/pot of P_2_O_5_ and 9.6 g/pot of K_2_O were used. Each treatment was grown in 20 pots. Each pot was 100 cm in height, 80 cm in length, and 60 cm in width and contained 650 kg of soil. The pots were rectangular trough planters instead of traditional pots. The potted plants were placed in the rain shelter, and the rain shelter was opened on sunny days. The rain shelter was built using a steel frame, which was electric, ventilated, and covered with cloth (could be moved back and forth on the steel frame).

### 2.3 Sampling and measurements

Sampling and measurements were conducted at mid-tillering (MT), panicle initiation (PI), heading (HD), and maturity (MA). Three plants of pot were used to measure root biomass, root oxidation activity, root total absorbing surface area, root active absorbing surface area, root bleeding rate, Z + ZR contents in roots, leaf photosynthetic rate, NSC accumulation in stems, and NR activity in leaves. Each treatment was repeated three times.

#### 2.3.1 Root dry weight

For each root sample, a sampling core was used to excavate a soil cube (20 cm × 20 cm × 20 cm) surrounding every individual hill. Such a cube holds approximately 95% of the entire biomass of the roots (Guan et al., 2022; Shotaro et al., 2023). A hydropneumatic elutriation device (Gillison’s Variety Fabrication, Benzonia, MI, USA) was used to cleanse the roots of every soil block. Separated shoots and roots were placed in paper sacks. The roots were dried in an oven at 75°C to constant weight and weighed.

#### 2.3.2 Root oxidation activity

The Liu et al. (2022) approach was used to measure the oxidation of alpha-naphthylamine (α-NA). Approximately 1 g of roots (on a fresh weight basis, cut into 1–2-cm segments) was preincubated for 10 minutes in 50 mL of 20 μmol/L α-NA test solution to avoid the rapid adsorption in the determination assay where roots were transferred to another 50 mL of 20 μmol/L α-NA test solution and incubated for 4 h after the preincubation step. Following the incubation period, the aliquots were filtered, and an aliquot of 2 mL of α-NA sample solution was treated with 10 ml of 0.1% sulfanilic acid (in 3% acetic acid) and 2 mL of 50 μmol/L NaNO_2_ and diluted to 25 mL with distilled water. The absorbance of the colored solution was determined at 530 nm using a UV spectrometer (7752G, Shanghai Yidian, Shanghai, China).

#### 2.3.3 Root bleeding rate

In accordance with Zhang et al. (2022), the root bleeding rate was determined. Each plant was cut at an internode that was 10 cm or so above the earth at 18:00. On the stem’s cutting edge, a pre-weighed Ziploc bag containing absorbent cotton was placed and secured with an elastic band. Each incline was protected from dust and vermin using a polyethylene sheet. At 6:00 a.m. the following day, the bleeding sap was gathered. The root bleeding rate was recorded and computed using the increase in cotton weight.

#### 2.3.4 Root total and active absorbing surface area

The methylene blue dyeing method was used to calculate the root total and active absorbing surface area (Zhang et al., 2019). The roots were thoroughly cleaned with distilled water, dried using absorbent paper, and then submerged for 1.5 minutes in a series of three beakers containing methylene blue solution. Back in the original beakers, the solution was permitted to drain naturally. Using an ultraviolet spectrophotometer (UV-2450, Shimadzu, Tokyo, Japan), the volume (V1, V2, and V3) of the solution in three beakers was calculated. The absorbance of the methylene blue solution diluted 10 times at a wavelength of 660 nm was recorded. Using the standard curve of the concentration (C′1, C′2, and C′3) of the methylene blue solution, the root total and active absorbing surface area were estimated.

Results were calculated based on the following equations:


$$\begin{array}{c} \text{Root total absorbing surface area }({\text{m}}^{2})=[(\text{C}-{\text{C}}^{\prime }1)\times \text{V}1]\times 1.1\\ +[(\text{C}-{\text{C}}^{\prime }2)\times \text{V}2]\times 1.1, \end{array}$$

$$\text{Root active absorbing surface area }({\text{m}}^{2})=[(\text{C}-{\text{C}}^{\prime }3)\times \text{V}3]\times 1.1,$$

where C is the original solution concentration (mg/mL), C′ is the leaching solution concentration, V is the solution volume, and 1, 2, and 3 are the number of beakers.

#### 2.3.5 Z + ZR contents in roots

Three hills from each treatment were used to freeze the roots, which were then maintained at −80°C. Liu et al. (2020) reported the method for extracting cytokinins (Z and ZR). Cytokinins were detected using the methods for liquid chromatography–tandem mass spectrometry (LC-MS/MS) with multiple reaction monitoring (MRM; Thermo Fisher Scientific, Waltham, MA, USA). Using a calibration curve with known standard amounts and the proportion of the cytokinins’ MRM transition summed area to that of their internal standards, cytokinins were quantified. The mixed standard solution of 5, 10, 25, 50, and 100 PPB concentration gradients was prepared using the external standard method, and the corresponding peak area was measured to obtain the standard curve. Thermo Fisher Scientific (Waltham, MA, USA) Xcalibur Data System was used for data collection and processing.

#### 2.3.6 NSC accumulation in stems

According to Junko et al. (2023), stems were taken out and crushed to figure out the content of NSC (starch and soluble sugar). Samples that had been dried in the oven were crushed through a 1-mm sieve after being reduced to a fine powder. The content of soluble sugar in the extract isomers of the 0.1-g powder sample was determined using an anthrone reagent. The starch content of the residue was determined using perchloric acid (9.36 mol/L). In colorimetric analysis, a UV-1800 spectrophotometer (Shimadzu, Tokyo, Japan) was used to evaluate the optical density at 620 nm. Using the anthrone reagent, the glucose produced during extraction was calculated. NSC concentration multiplied by stem biomass results in a value for the accumulation of NSC in stems (g/pot).

#### 2.3.7 Leaf photosynthetic rate

The leaf photosynthetic rate was determined using the Li-Cor 6400 portable photosynthesizer (Li-Cor, Lincoln, NE, USA). The measurement was performed between 9:00 and 11:00 a.m. for three randomly selected healthy leaves, while the canopy was exposed to 1,000 μmol m^−2^ s^−1^ of UV light that was involved in photosynthetic processes.

#### 2.3.8 NR activity in leaves

NR activity in leaves was determined by Chen et al. (2022). Using a phosphate buffer with a pH of 7.5, composed of K_2_HPO_4_ and KH_2_PO_4_, 0.0372 g/L EDTA, and 0.1211 g/L cysteine, the extraction solution was prepared. A volume of 0.4 mL of liquid was collected, and potassium nitrate buffer and 0.2 mL of phosphate buffer control group were added at 25°C and reacted for 30 minutes. A volume of 1.0 mL of 1% sulfonamide solution and 1.0 mL of 0.2% naphthylamine solution was added and centrifuged at 4,500 rpm for 15 minutes, and then the upper solution’s absorbance was measured at 540 nm.

#### 2.3.9 Harvest

Grain yield was determined from three plants per pot and adjusted to 14% moisture. Yield components, i.e., the number of panicles per pot, spikelet number per panicle, percentage of filled grains, and grain weight, were determined from plants in three pots sampled randomly. The percentage of filled grains was calculated by dividing the number of filled grains (specific gravity ≥1.06 g/cm^3^) by the total number of spikelets.


$$\text{The grain yield}\;=\frac{14\%}{\text{moisture content }\left(\%\right)}\;*\text{the grain yield with moisture.}$$

Moisture content was determined using a moisture determination instrument. The grain yield with moisture was weighed using a balance (JE3003, Shanghai Shangyi, Shanghai, China) after harvest.

### 2.4 Statistical analysis

To test if grain yield and its components (panicles per pot, spikelets per panicle, total spikelets, filled grains, and grain weight) varied with year, variety, and treatment, a series of three-way analysis of variance (ANOVA) were conducted followed by Tukey’s honestly significant difference (HSD) tests with year, variety, and treatment as fixed factors and each grain yield and its components as responses. All data were checked for normality and homoscedasticity using the Shapiro–Wilk test and Bartlett’s test, respectively. When these assumptions were not met, non-parametric Kruskal–Wallis tests were used. Additionally, Pearson’s correlation coefficients were used to explore the relationship between grain yield and root morphophysiology, as well as aboveground physiological indexes. All statistical tests were performed using SPSS 21.0 (SPSS Inc., Chicago, IL, USA). Statistical significance was established at p-values<0.05. The figures were drawn using SigmaPlot 11.0 (SPSS Inc., Point Richmond, CA, USA) and R 4.0.2 (R Core Team).

## 3 Results

### 3.1 Grain yield and its components

The grain yield of Hyou 518 and Hanyou 73 increased with the increase of NFAR under the same irrigation regime (**Table 1**). The panicles, spikelet number per panicle, and total spikelets increased with the increase of NFAR under the same irrigation regime, and the filled grains and grain weight decreased with the increase of NFAR. Under the same NFAR, compared with continuous flooding cultivation, water stress significantly reduced the grain yield of Hyou 518 by 7.1%–15.1%, and the significant decrease in total spikelets was the main reason for the decrease in grain yield. However, under the same NFAR, different irrigation regimes had no significant effect on the grain yield of Hanyou 73. The grain yield of Hanyou 73 under MN-60% was basically higher than that of Hyou 518 under other treatments (**Table 1**).

**Table 1:** Yield and its components of two varieties under different irrigation regimes and nitrogen fertilizer application rates.

Year	Variety	Treatments	Grain yield (g/pot)	Panicles per pot	Spikelets per panicle	Total spikelets (×10^3^ pot^−1^)	Filled grains (%)	Grain weight (mg)
2019	Hyou 518	0N-60%	81.6 e	49.1 c	84.0 d	4.12 f	81.8 a	24.2 a
		0N-100%	91.2 d	51.2 c	88.6 c	4.54 e	82.4 a	24.4 a
		MN-60%	172.8 c	85.7 b	112.1 b	9.61 d	75.9 b	23.7 b
		MN-100%	190.4 ab	87.2 b	117.9 a	10.3 c	77.8 b	23.8 b
		HN-60%	183.7 b	95.4 a	118.4 a	11.3 b	70.7 c	23.0 c
		HN-100%	197.8 a	97.8 a	122.1 a	11.9 a	71.1 c	23.3 c
	Hanyou 73	0N-60%	95.4 c	40.9 c	105.1 c	4.30 c	84.4 a	26.3 a
		0N-100%	98.9 c	41.1 c	107.7 c	4.43 c	84.6 a	26.4 a
		MN-60%	195.1 b	64.8 b	144.4 b	9.36 b	80.8 b	25.8 b
		MN-100%	201.9 ab	65.3 b	147.4 b	9.63 b	81.3 ab	25.8 b
		HN-60%	198.4 ab	69.8 a	157.3 a	11.0 a	71.7 c	25.2 c
		HN-100%	206.4 a	70.6 a	160.9 a	11.4 a	71.8 c	25.3 c
2020	Hyou 518	0N-60%	78.4 e	50.5 d	81.8 d	4.13 f	78.4 a	24.2 a
		0N-100%	90.4 d	51.6 d	89.8 c	4.63 e	80.3 a	24.3 a
		MN-60%	165.5 c	82.4 c	116.5 b	9.59 d	73.1 bc	23.6 b
		MN-100%	194.9 a	88.8 b	119.8 ab	10.6 c	76.8 b	23.9 ab
		HN-60%	182.9 b	95.6 a	120.2 ab	11.5 b	68.9 d	23.1 c
		HN-100%	202.5 a	98.9 a	124.1 a	12.3 a	70.5 cd	23.4 bc
	Hanyou 73	0N-60%	92.7 c	40.1 c	104.6 d	4.20 c	85.3 a	25.9 ab
		0N-100%	95.0 c	40.7 c	103.5 d	4.21 c	86.1 a	26.2 a
		MN-60%	187.9 b	63.5 b	145.4 c	9.23 b	79.6 b	25.6 b
		MN-100%	197.5 ab	64.8 b	148.1 bc	9.59 b	79.8 b	25.8 ab
		HN-60%	198.5 ab	70.5 a	154.7 ab	10.9 a	72.4 c	25.1 c
		HN-100%	205.7 a	71.2 a	158.1 a	11.3 a	72.8 c	25.1 c
Analysis of variance								
Year (Y)			NS	NS	NS	NS	NS	NS
Variety (V)			9.94^**^	379.5^**^	740.7^**^	7.95^**^	44.0^**^	206.6^**^
Treatment (T)			167.4^**^	186.8^**^	247.5^**^	280.6^**^	60.7^**^	3.26^*^
Y × V			NS	NS	NS	NS	NS	NS
Y × T			NS	NS	NS	NS	3.86^**^	NS
V × T			NS	2.87^*^	11.7^**^	NS	4.34^**^	NS
Y × V × T			4.30^**^	4.48^**^	NS	4.82^**^	NS	NS

NS indicates statistical significance at *p* > 0.05 within same variety in the same year. ^*^ and ^**^ represent statistical significance at *p*< 0.05 and *p*< 0.01, respectively. 0N, MN, and HN represent no nitrogen applied, medium nitrogen applied (15.6 g/pot), and high nitrogen applied (31.2 g/pot), respectively. 100% and 60% represent continuous flooding cultivation and 60% of continuous flooding cultivation, respectively.

### 3.2 Root dry weight

**Figure 1:**
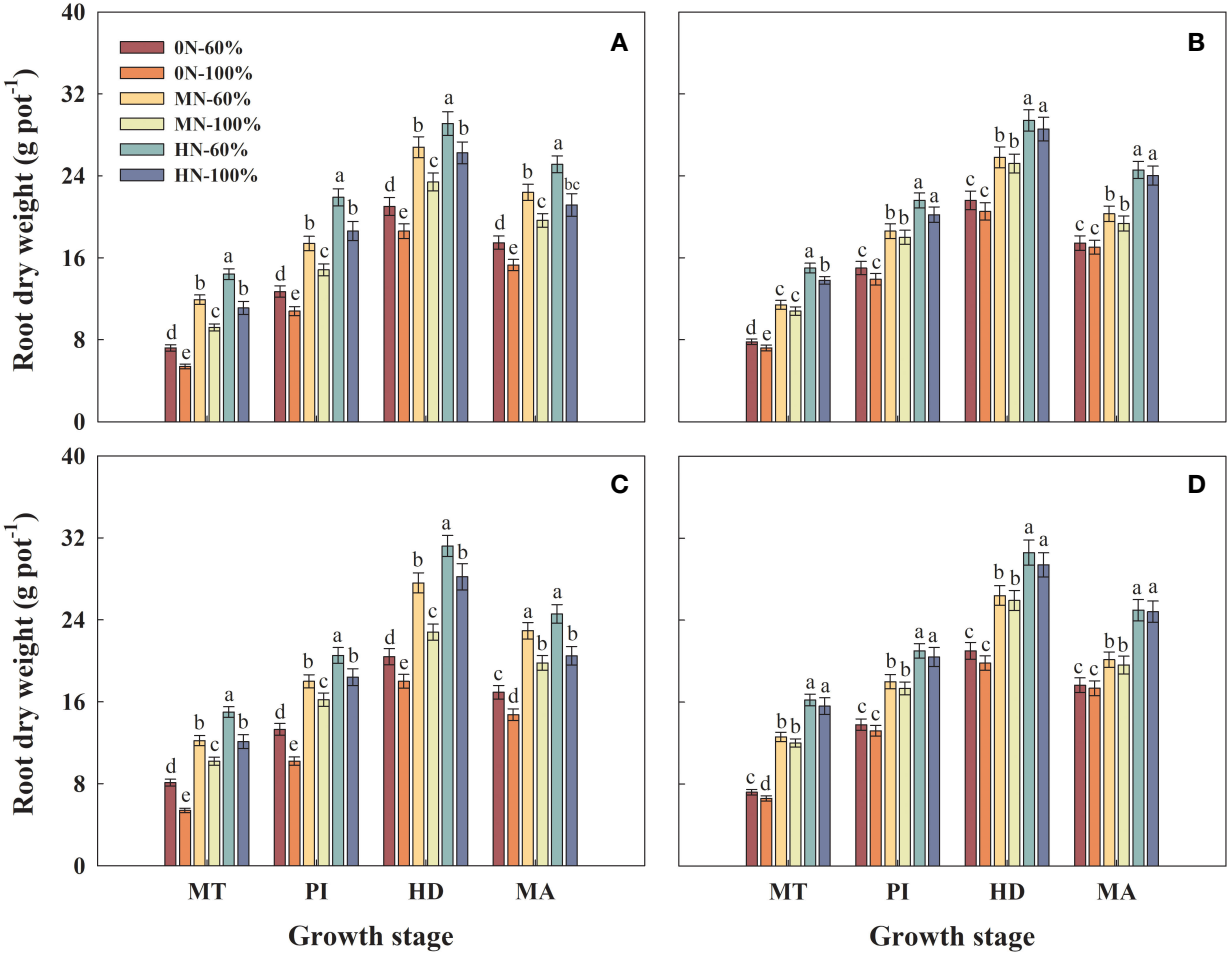
Root dry weight of Hyou 518 **(A, C)** and Hanyou 73 **(B, D)** under different irrigation regimes and nitrogen fertilizer application rates in 2019 **(A, B)** and 2020 **(C, D)**. 0N, MN, and HN represent no nitrogen applied, medium nitrogen applied (15.6 g/pot), and high nitrogen applied (31.2 g/pot), respectively. 100% and 60% represent continuous flooding cultivation and 60% of continuous flooding cultivation, respectively. MT, PI, HD, and MA represent mid-tillering, panicle initiation, heading, and maturity, respectively. Different letters above the column indicate statistical significance at *p*< 0.05 level within the same stage. Vertical bars represent ± SE of the mean. The SE was calculated across three replications for each year.

The root dry weight of Hyou 518 and Hanyou 73 increased significantly with the increase of NFAR within the same growth stage under the same irrigation regime (**Figure 1**). The root dry weight of Hyou 518 significantly increased at key growth stages (MT, PI, HD, and MA) in 0N-60%, MN-60%, and HN-60%, compared with 0N-100%, MN-100%, and HN-100% treatments, respectively (**Figures 1A, C**). Under the same NFAR, different irrigation regimes had little effect on Hanyou 73 (**Figures 1B, D**).

### 3.3 Root oxidation activity

**Figure 2:**
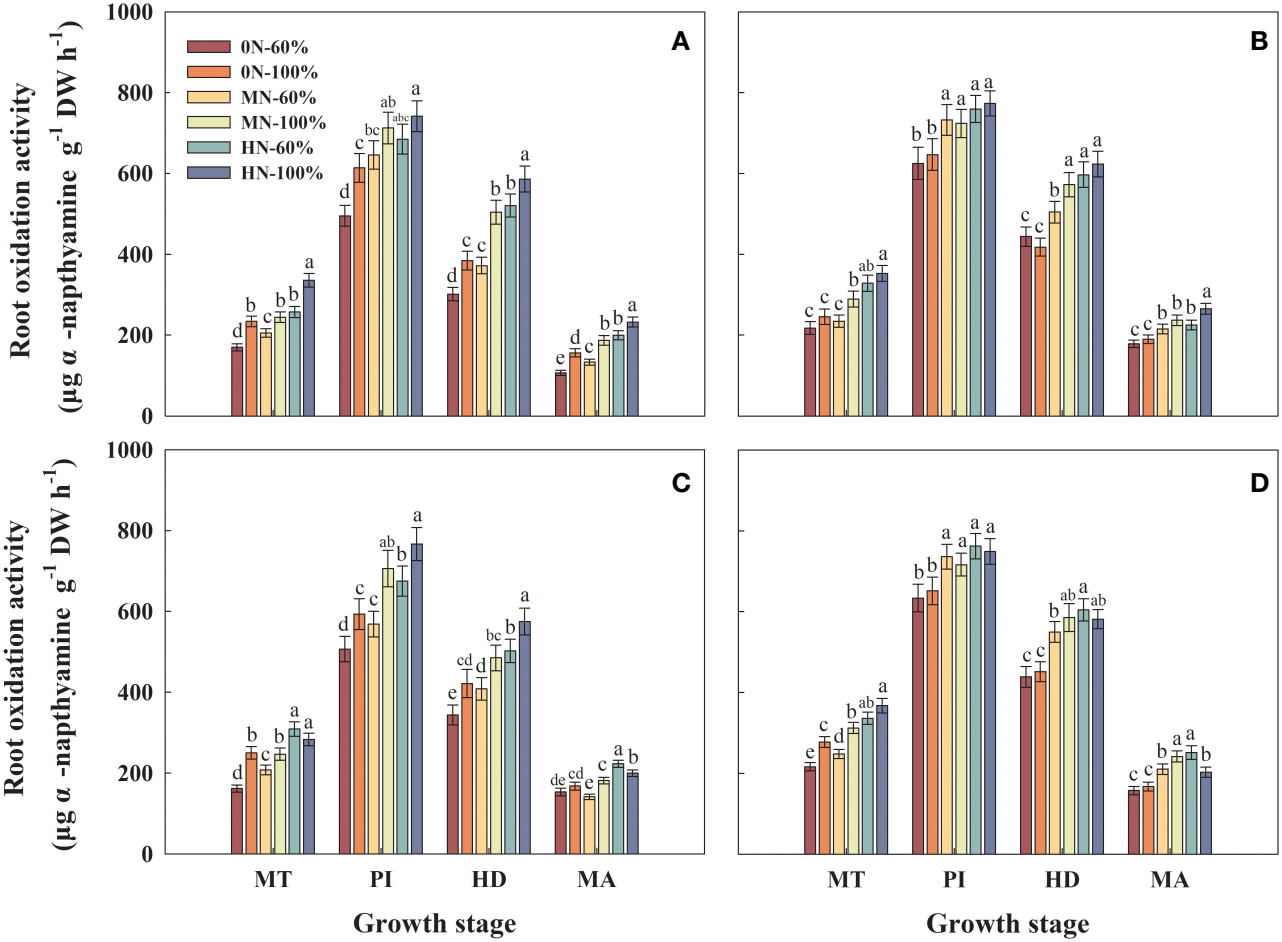
Root oxidation activity of Hyou 518 **(A, C)** and Hanyou 73 **(B, D)** under different irrigation regimes and nitrogen fertilizer application rates in 2019 **(A, B)** and 2020 **(C, D)**. 0N, MN, and HN represent no nitrogen applied, medium nitrogen applied (15.6 g/pot), and high nitrogen applied (31.2 g/pot), respectively. 100% and 60% represent continuous flooding cultivation and 60% of continuous flooding cultivation, respectively. MT, PI, HD, and MA represent mid-tillering, panicle initiation, heading, and maturity, respectively. Different letters above the column indicate statistical significance at *p*< 0.05 level within the same stage. Vertical bars represent ± SE of the mean. The SE was calculated across three replications for each year.

The root oxidation activity of Hyou 518 under 0N-60%, MN-60%, and HN-60% decreased by 17.1%–23.9%, 9.4%–24.1%, and 7.7%–13.6%, respectively, compared with 0N-100%, MN-100%, and HN-100% at PI, respectively (**Figures 2A, C**). Under the same NFAR in the same growth stage, different irrigation regimes had little effect on the root oxidation activity of Hanyou 73 (**Figures 2B, D**).

### 3.4 Root bleeding rate

**Figure 3:**
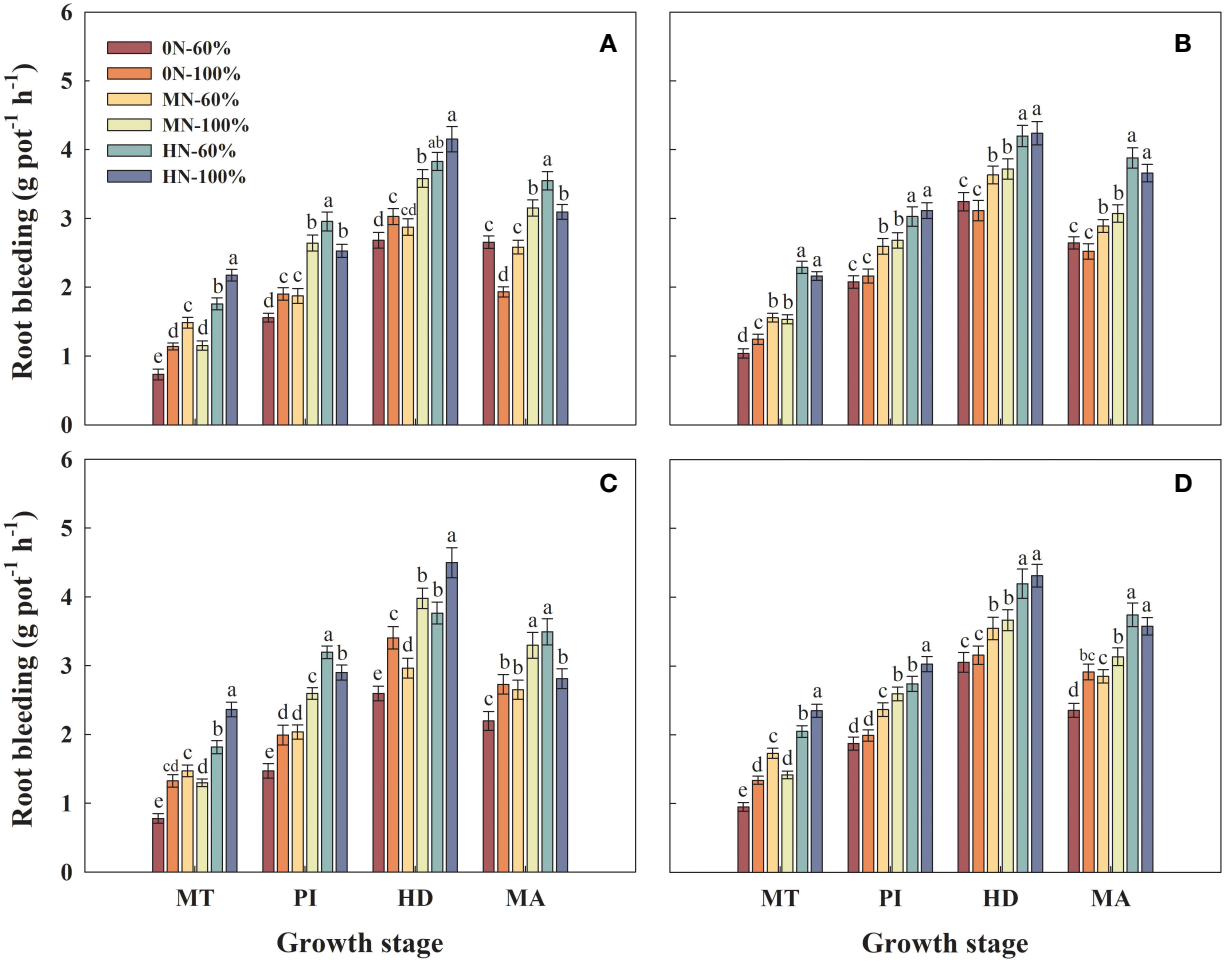
Root bleeding rate of Hyou 518 **(A, C)** and Hanyou 73 **(B, D)** under different irrigation regimes and nitrogen fertilizer application rates in 2019 **(A, B)** and 2020 **(C, D)**. 0N, MN, and HN represent no nitrogen applied, medium nitrogen applied (15.6 g/pot), and high nitrogen applied (31.2 g/pot), respectively. 100% and 60% represent continuous flooding cultivation and 60% of continuous flooding cultivation, respectively. MT, PI, HD, and MA represent mid-tillering, panicle initiation, heading, and maturity, respectively. Different letters above the column indicate statistical significance at *p*< 0.05 level within the same stage. Vertical bars represent ± SE of the mean. The SE was calculated across three replications for each year.

In the same growth stage, the root bleeding rate of the two varieties increased with the increase of NFAR under the same irrigation regime. Under the same NFAR, the root bleeding rate of Hyou 518 under MN-100% increased by 27.5%–41.1% and 24.6%–34.3%, compared with MN-60% at PI and HD, respectively (**Figures 3A, C**). There were basically no significant differences in the root bleeding rate of Hanyou 73 under the two irrigation regimes during the same growth stage (**Figures 3B, D**).

### 3.5 Root total absorbing surface area

**Figure 4:**
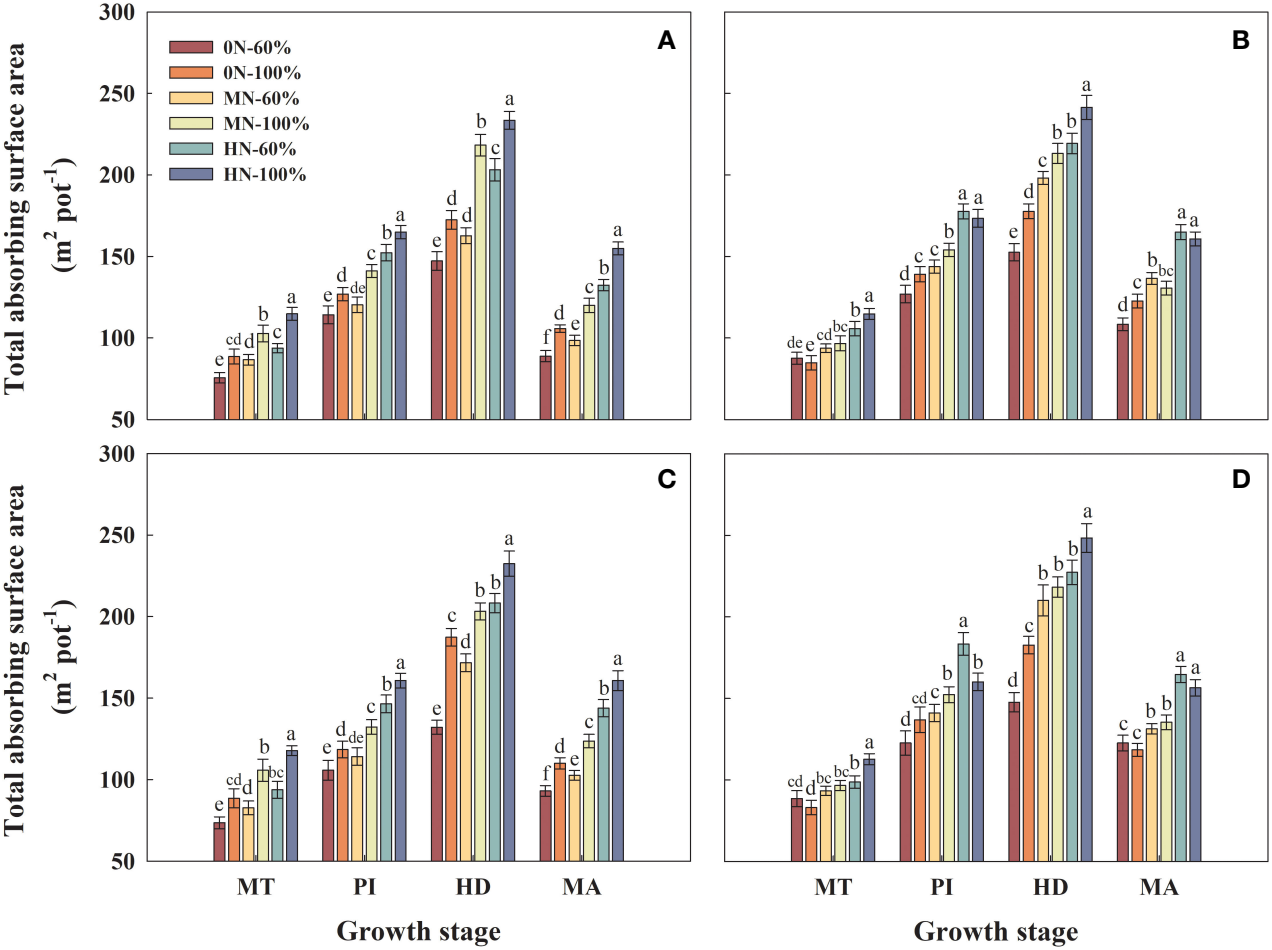
Root total absorbing surface area of Hyou 518 **(A, C)** and Hanyou 73 **(B, D)** under different irrigation regimes and nitrogen fertilizer application rates in 2019 **(A, B)** and 2020 **(C, D)**. 0N, MN, and HN represent no nitrogen applied, medium nitrogen applied (15.6 g/pot), and high nitrogen applied (31.2 g/pot), respectively. 100% and 60% represent continuous flooding cultivation and 60% of continuous flooding cultivation, respectively. MT, PI, HD, and MA represent mid-tillering, panicle initiation, heading, and maturity, respectively. Different letters above the column indicate statistical significance at *p*< 0.05 level within the same stage. Vertical bars represent ± SE of the mean. The SE was calculated across three replications for each year.

Compared with 0N-60% treatment, the root total absorbing surface area of Hyou 518 under MN-60% and HN-60% treatments at HD increased by 10.6%–29.9% and 38.0%–57.6%, respectively (**Figures 4A, C**). Hyou 518 significantly increased by 17.1%–41.8%, 18.4%–34.1%, and 11.6%–14.9% under 0N-100%, MN-100%, and HM-100%, respectively, compared with 0N-60%, MN-60%, and HM-60%, respectively (**Figures 4A, C**). The root total absorbing surface area of Hanyou 73 had a great response to NFAR but had little effect on irrigation regimes (**Figures 4B, D**).

### 3.6 Root activity absorbing surface area

**Figure 5:**
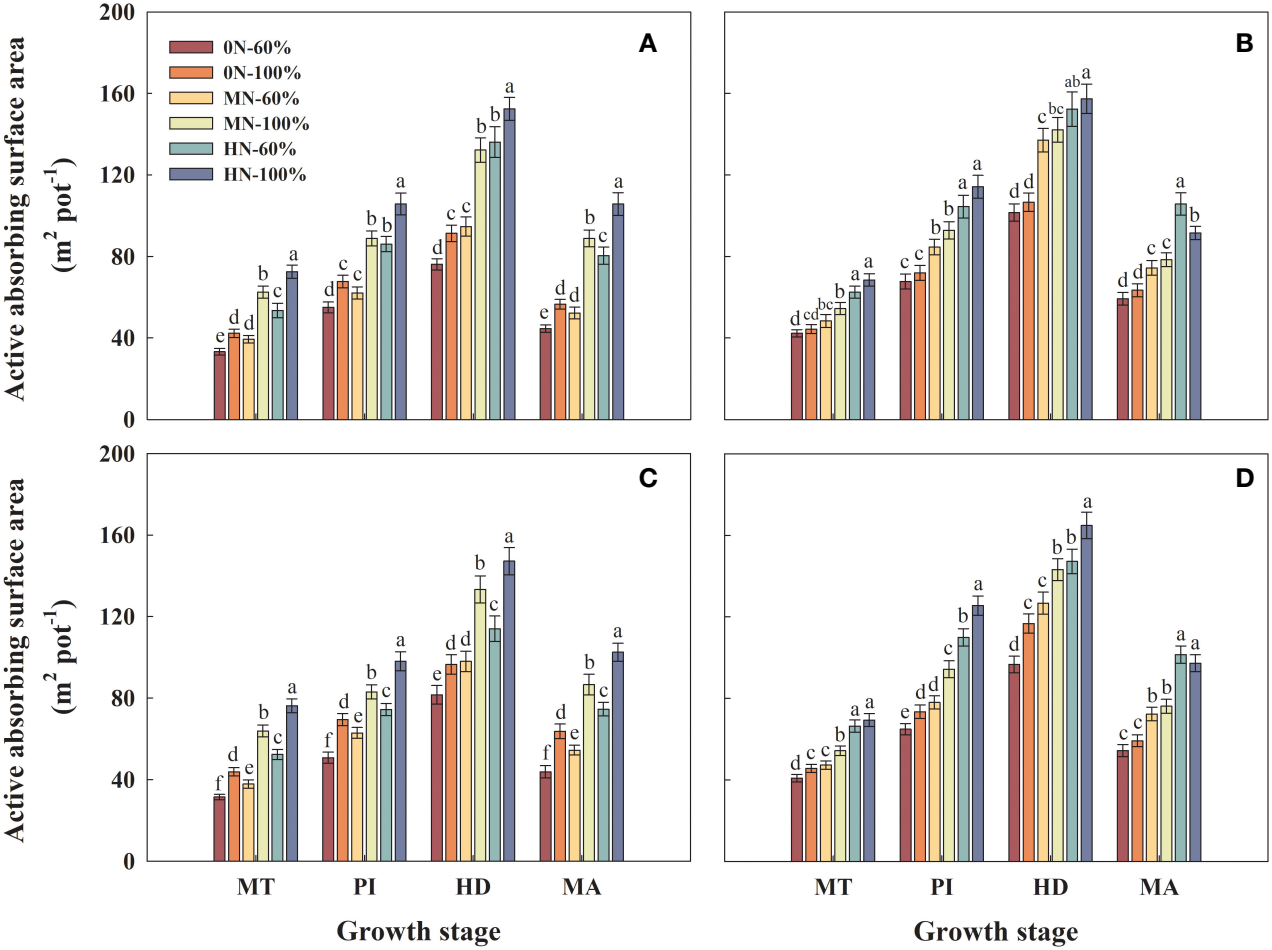
Root active absorbing surface area in roots of Hyou 518 **(A, C)** and Hanyou 73 **(B, D)** under different irrigation regimes and nitrogen fertilizer application rates in 2019 **(A, B)** and 2020 **(C, D)**. 0N, MN, and HN represent no nitrogen applied, medium nitrogen applied (15.6 g/pot), and high nitrogen applied (31.2 g/pot), respectively. 100% and 60% represent continuous flooding cultivation and 60% of continuous flooding cultivation, respectively. MT, PI, HD, and MA represent mid-tillering, panicle initiation, heading, and maturity, respectively. Different letters above the column indicate statistical significance at *p*< 0.05 level within the same stage. Vertical bars represent ± SE of the mean. The SE was calculated across three replications for each year.

At the same nitrogen level, the active absorption surface area of the two varieties increased significantly with the increase of irrigation amount, which had a greater effect on Hyou 518 compared with Hanyou 73. Compared with MN-60% treatment, the root activity absorbing surface area of Hyou 518 and Hanyou 73 under MN-100% treatment at HD increased by 36.0%–39.6% and 3.70%–13.0%, respectively (**Figure 5**). The trend of root active absorbing surface area was basically consistent with the root total absorbing surface area (**Figure 5**).

### 3.7 Z + ZR contents in roots

**Figure 6:**
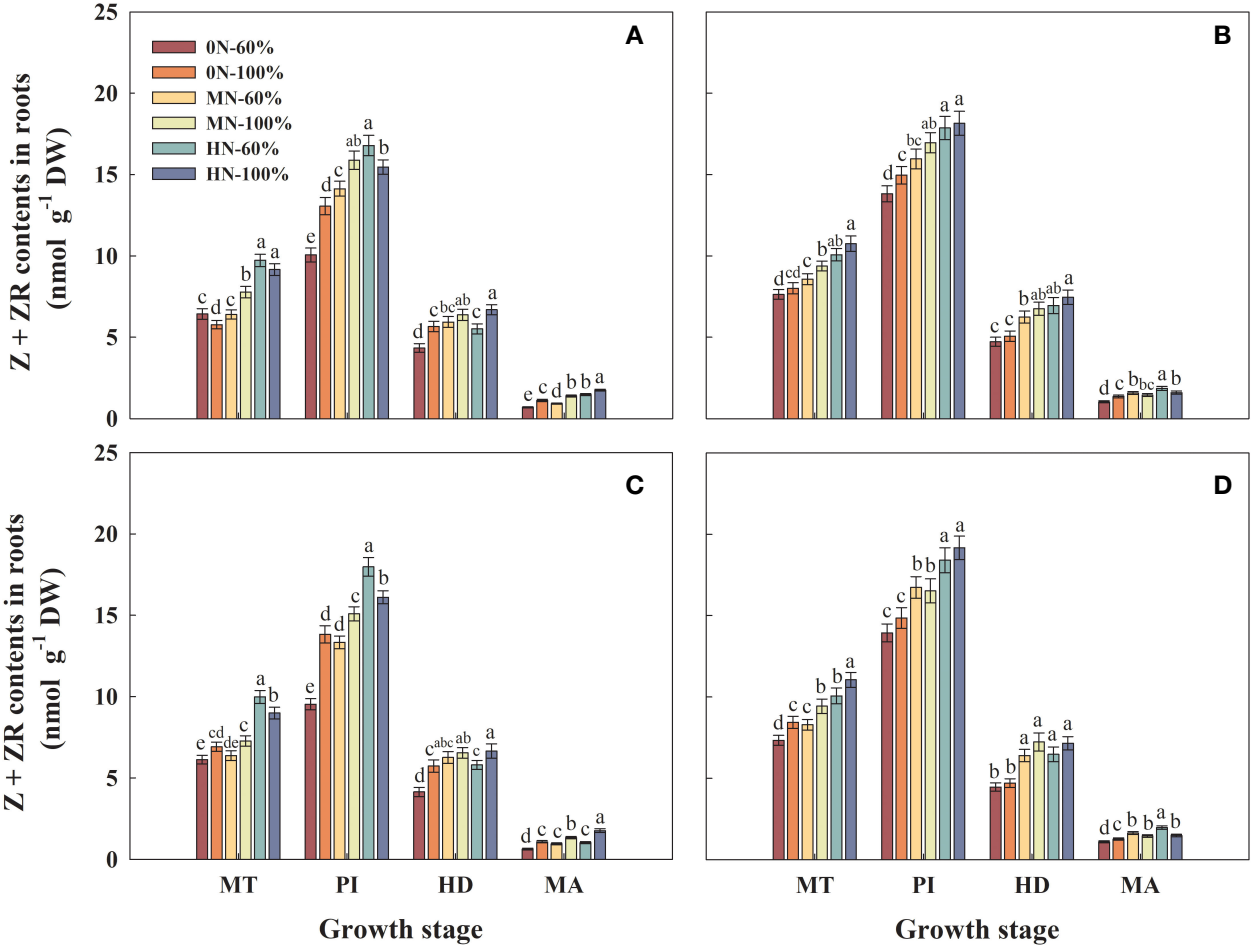
Zeatin (Z) + zeatin riboside (ZR) contents in roots of Hyou 518 **(A, C)** and Hanyou 73 **(B, D)** under different irrigation regimes and nitrogen fertilizer application rates in 2019 **(A, B)** and 2020 **(C, D)**. 0N, MN, and HN represent no nitrogen applied, medium nitrogen applied (15.6 g/pot), and high nitrogen applied (31.2 g/pot), respectively. 100% and 60% represent continuous flooding cultivation and 60% of continuous flooding cultivation, respectively. MT, PI, HD, and MA represent mid-tillering, panicle initiation, heading, and maturity, respectively. Different letters above the column indicate statistical significance at *p*< 0.05 level within the same stage. Vertical bars represent ± SE of the mean. The SE was calculated across three replications for each year.

The Z + ZR contents in roots of Hyou 518 under 0N-100% and MN-100% increased by 29.8%–45.0% and 12.4%–13.1%, respectively, compared with 0N-60% and MN-60%, respectively. Compared with HN-60%, Z + ZR contents in roots of Hyou 518 decreased by 7.9%–10.4% under HN-100% (**Figures 6A, C**). Under the same NFAR, different irrigation regimes had little effect on Z + ZR contents in the roots of Hanyou 73 (**Figures 6B, D**).

### 3.8 Leaf photosynthetic rate

**Figure 7:**
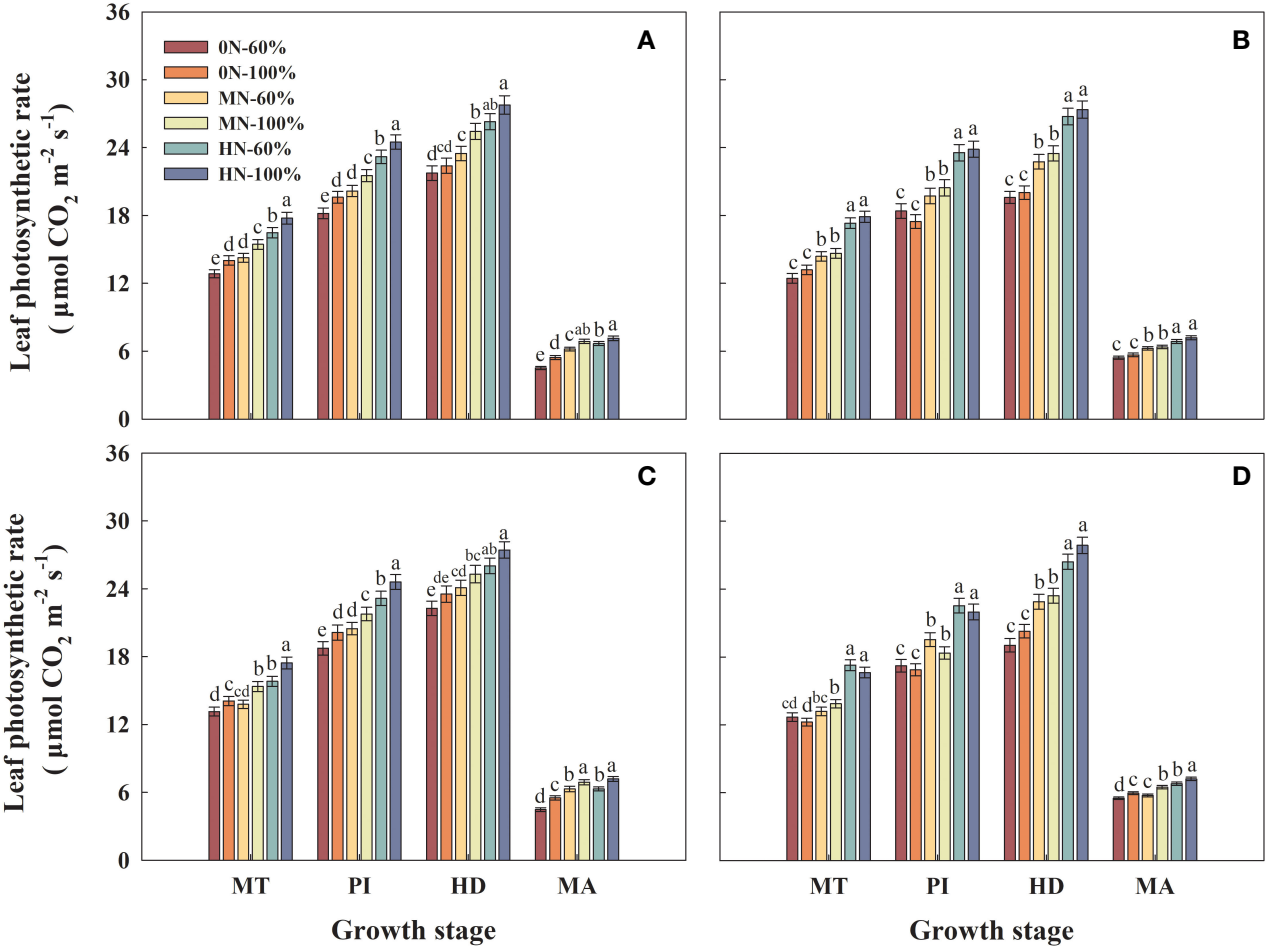
Leaf photosynthetic rate of Hyou 518 **(A, C)** and Hanyou 73 **(B, D)** under different irrigation regimes and nitrogen fertilizer application rates in 2019 **(A, B)** and 2020 **(C, D)**. 0N, MN, and HN represent no nitrogen applied, medium nitrogen applied (15.6 g/pot), and high nitrogen applied (31.2 g/pot), respectively. 100% and 60% represent continuous flooding cultivation and 60% of continuous flooding cultivation, respectively. MT, PI, HD, and MA represent mid-tillering, panicle initiation, heading, and maturity, respectively. Different letters above the column indicate statistical significance at *p*< 0.05 level within the same stage. Vertical bars represent ± SE of the mean. The SE was calculated across three replications for each year.

Under the same irrigation regime, the leaf photosynthetic rate of Hyou 518 and Hanyou 73 increased with the increase of NFAR during the same growth stage (**Figure 7**). Compared with that under MN-60% treatment, the leaf photosynthetic rate of Hyou 518 and Hanyou 73 under HN-60% treatment at PI increased by 13.0%–15.0% and 15.4%–19.4%, respectively (**Figure 7**). Under the same NFAR, increasing the irrigation amount could increase the leaf photosynthetic rate of Hyou 518 but had little effect in Hanyou 73 (**Figure 7**).

### 3.9 NSC accumulation in stems

**Figure 8:**
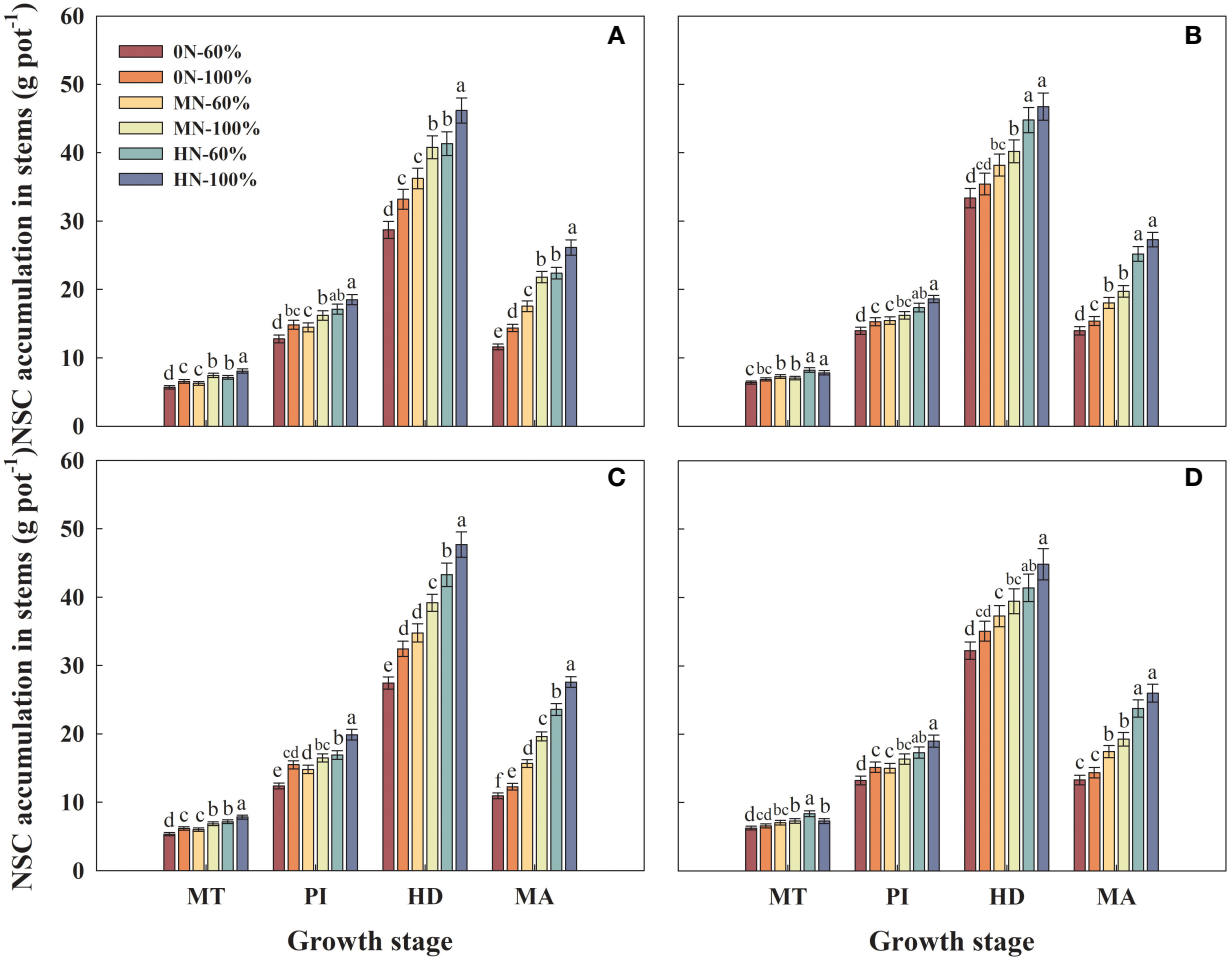
Non-structural carbohydrate (NSC) accumulation in stems of Hyou 518 **(A, C)** and Hanyou 73 **(B, D)** under different irrigation regimes and nitrogen fertilizer application rates in 2019 **(A, B)** and 2020 **(C, D)**. 0N, MN, and HN represent no nitrogen applied, medium nitrogen applied (15.6 g/pot), and high nitrogen applied (31.2 g/pot), respectively. 100% and 60% represent continuous flooding cultivation and 60% of continuous flooding cultivation, respectively. MT, PI, HD, and MA represent mid-tillering, panicle initiation, heading, and maturity, respectively. Different letters above the column indicate statistical significance at *p*< 0.05 level within the same stage. Vertical bars represent ± SE of the mean. The SE was calculated across three replications for each year.

Compared with that under MN-60% treatment, the NSC accumulation in stems of Hyou 518 and Hanyou 73 under HN-60% treatment at HD increased by 14.1%–24.5% and 11.1%–17.3%, respectively (**Figure 8**). Under the same NFAR, the NSC accumulation in stems of Hyou 518 and Hanyou 73 increased with the increase of irrigation amount at HD. The increased rate of NSC accumulation in stems of Hyou 518 was significantly higher than that of Hanyou 73 (**Figure 8**).

### 3.10 NR activity in leaves

**Figure 9:**
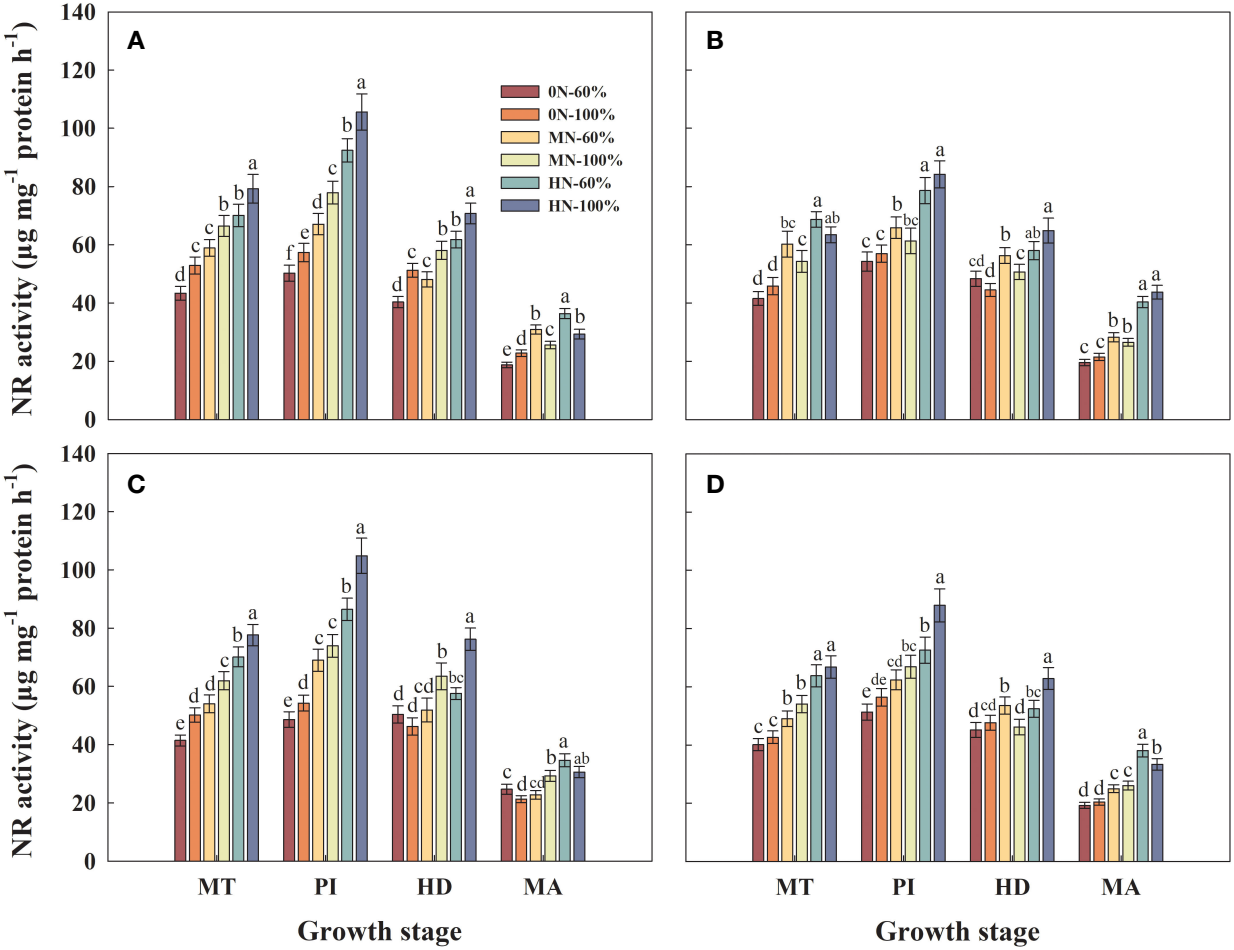
Nitrate reductase (NR) activity in leaves of Hyou 518 **(A, C)** and Hanyou 73 **(B, D)** under different irrigation regimes and nitrogen fertilizer application rates in 2019 **(A, B)** and 2020 **(C, D)**. 0N, MN, and HN represent no nitrogen applied, medium nitrogen applied (15.6 g/pot), and high nitrogen applied (31.2 g/pot), respectively. 100% and 60% represent continuous flooding cultivation and 60% of continuous flooding cultivation, respectively. MT, PI, HD, and MA represent mid-tillering, panicle initiation, heading, and maturity, respectively. Different letters above the column indicate statistical significance at *p*< 0.05 level within the same stage. Vertical bars represent ± SE of the mean. The SE was calculated across three replications for each year.

Under the same irrigation regime, the NR activity in the leaves of Hyou 518 and Hanyou 73 increased with the increase of NFAR at PI (**Figure 9**). The Z + ZR contents in roots of Hyou 518 with 0N-100%, MN-100%, and HN-100% were increased by 11.6%–14.2%, 7.1%–16.1%, and 14.2%–21.3% at PI, respectively, compared with 0N-60%, MN-60%, and HN-60%, respectively (**Figures 9A, C**). At the same NFAR, different irrigation regimes had little effect on NR activity in leaves of Hanyou 73 at MT, PI, HD, and MA (**Figures 9B, D**).

### 3.11 Correlation analysis

The grain yield of Hyou 518 was basically positively correlated with NSC accumulation in stems, leaf photosynthetic rate, and NR activity in leaves in the key growth stages (MT, PI, HD, and MA). The grain yield of Hanyou 73 correlated significantly positively with root dry weight, root oxidation activity, root bleeding rate, root total absorbing surface area, root active absorbing surface area, and Z + ZR contents in roots at PI and HD, and the correlation was basically higher than that of Hyou 518.

## 4 Discussion

### 4.1 Effect of different irrigation regimes and NFAR on grain yield in WDR

Water and nitrogen management plays an essential role in high-yield and water-efficient irrigation production in rice (Sun et al., 2012). Previous studies have found that moderate water stress increases yield by 2.74% on average, compared with flooded irrigation (Yang et al., 2017). Moreover, the yield increase was the best under moderate water stress coupled with medium NFAR. However, rice yield would decrease significantly under severe water stress and high NFAR (Xu et al., 2018). The significant decrease in the number of panicles per unit of space is the main reason for the low grain yield under severe water stress (Cao et al., 2017). Although the high NFAR increased the total spikelets and the number of spikelets per panicle in rice, it significantly decreased the filled grains and grain weight, thus reducing rice yield (Peng et al., 2021). In this study, the grain yield of Hyou 518 under water stress was decreased to different degrees in the same NFAR, compared with continuous flooding cultivation (**Table 1**). The decrease in total spikelets was the main reason for the decrease in grain yield. However, there were no significant effects on the grain yield of Hanyou 73 under the two irrigation regimes. These results indicate that WDR can withstand drought better than rice at the same NFAR. Hanyou 73 could still obtain a higher yield under the interaction treatment of moderate NFAR and water stress. Therefore, an appropriate reduction of irrigation amount (especially) and NFAR could still maintain a high yield in WDR.

### 4.2 Physiological mechanism of different irrigation regimes and NFAR on grain yield in WDR

**Figure 10:**
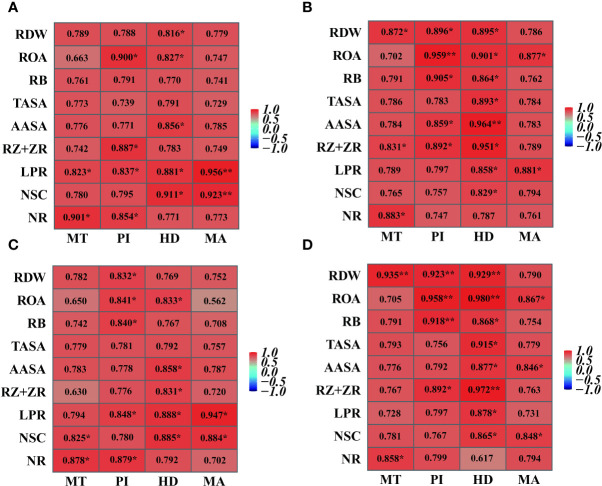
Correlation between root morphophysiological indexes and grain yield of Hyou 518 **(A, C)** and Hanyou 73 **(B, D)** under different irrigation regimes and nitrogen fertilizer application rates in 2019 **(A, B)** and 2020 **(C, D)**. MT, PI, HD, and MA represent mid-tillering, panicle initiation, heading, and maturity, respectively. RDW, ROA, RB, TASA, AASA, RZ + ZR, LPR, NSC, and NR represent root dry weight, root oxidation activity, root bleeding rate, root total absorbing surface area, root active absorbing surface area, zeatin + zeatin riboside contents in roots, leaf photosynthetic rate, non-structural carbohydrate accumulation in stems, and nitrate reductase activity in leaves, respectively. ^*^ and ^**^ indicate significant correlations at *p*< 0.05 and *p*< 0.01, respectively.

NR is a regulatory and rate-limiting enzyme in nitrate nitrogen assimilation (Han et al., 2022), and strong NR activity in leaves could increase the leaf photosynthetic rate and thus increase rice yield (Wu et al., 2018). NSCs such as starch, fructan, and sucrose are important substances for plant survival and metabolism (Liu et al., 2021). The NSC accumulation in stems was directly associated with the formation of rice yield (Wang et al., 2016). Previous research has demonstrated that appropriate NFAR could improve water and nutrient uptake by roots, leaf photosynthetic rate, NR activity in leaves, and NSC accumulation in stems and thus improve rice production (Ju et al., 2015). The results of this study were consistent in that increasing nitrogen fertilizer could synergistically increase the NR, NSC, and leaf photosynthetic rate of the two varieties. However, compared with continuous flooding cultivation, water stress can cause the above indexes to decrease, and the effect on Hyou 518 is greater than that on Hanyou 73 (**Figures 7**–**9**). Correlation analysis also found that the grain yield of Hyou 518 was significantly positively correlated with leaf photosynthetic rate, NR activity in leaves, and NSC accumulation in stems at PI and HD, and the correlation was higher than that of Hanyou 73 (**Figure 10**). These results indicate that compared with WDR, the improvement of aboveground physiological indexes is the reason for the high yield of Hyou 518 under the conditions of high NFAR and high irrigation amount.

Morphology and physiology of the aboveground part are closely related to root growth and development (Céline et al., 2010; Okami et al., 2012). Good aboveground parts could promote root growth (Yang et al., 2012). There was a significant relationship between root dry weight and grain yield at HD and MA (Yang et al., 2012). Root dry weight was one of the most pivotal morphological traits of roots, and the increase of root biomass was crucial to the improvement of rice yield (Zhang et al., 2019). Previous studies have observed that rice root biomass increases with the increase of NFAR (Liu et al., 2018) but decreases with the increase of field irrigation amount (Chu et al., 2014; Chen et al., 2019). The results of this study showed that the root dry weight at HD was significantly positively correlated with the grain yield in the two varieties, and the correlation of Hanyou 73 was significantly higher than that of Hyou 518 (**Figure 10**). The results showed that the improvement of root dry weight was the key root basis for the high yield of WDR under medium NFAR and low irrigation amount.

Root oxidation activity, root total absorbing surface area, root active absorbing surface area, and root bleeding rate are golden indicators of root activity (Yang et al., 2004). Increases in root activity are often strongly correlated with a high grain yield (Xu et al., 2015a). Some studies have observed that increased NFAR could alleviate the adverse effects of reduced soil water content on root activity and alleviate yield loss (Peng et al., 2017). The results of this study showed that under low nitrogen conditions, increasing NFAR could improve the root vitality of the two varieties and reduce the yield loss caused by water stress; this is consistent with the results of the above research. However, this feature was not obvious under high nitrogen conditions (**Table 1**; **Figure 2**). There were also studies that showed that high NFAR would inhibit root activity and aggravate the damage caused by water deficit in rice (Zhang et al., 2018). In this study, the increase of NFAR contributed to the increase of root activity and yield of the two varieties under the same water stress, which was inconsistent with the results of the above study.

Z + ZR contents in roots play a key role in encouraging endosperm cell proliferation, postponing plant senescence, and improving leaf photosynthetic rate (Yang et al., 2007; Xu et al., 2018). Increasing the Z + ZR contents at PI was beneficial to promote spikelet development and rice yield (Liu et al., 2020). In this study, it was observed that increasing the NFAR increased the root activity, Z + ZR contents in roots, and grain yield of both varieties under the same irrigation regime (**Figures 2**–**6**). Compared with continuous flooding cultivation, water stress reduced root activity and grain yield of Hyou 518 under the same NFAR but had less effects on root activity and grain yield of Hanyou 73. The correlation analysis also showed that there was a significant positive relationship between root activity and grain yield of Hanyou 73 at PI and HD (**Figure 10**). Therefore, the improvement of root morphology and physiology (root dry weight, root oxidation activity, root bleeding rate, root total absorbing surface area, root active absorbing surface area, and Z + ZR contents in roots) was a pivotal factor for WDR to maintain stable yield under water stress and moderate NFAR. Hormones such as indole-3-acetic and abscisic acid in roots also affect rice yield formation. For example, increasing the abscisic acid content and reducing the content of indole-3-acetic in roots during the grain-filling stage were beneficial to promote grain filling in rice (Xu et al., 2018). Therefore, the mechanisms of the interaction between plant hormones on rice yield remain to be further studied.

## 5 Conclusion

WDR is a new type of cultivated rice variety bred by integrating the excellent characteristics of modern rice and upland rice, which is conducive to promoting the development of resource-saving and environment-friendly agriculture in China. However, the effects and mechanisms of irrigation and nitrogen on the yield in WDR were still unclear. In this study, Hyou 518(paddy rice) had the highest yield under high NFAR and high irrigation amount. However, whether water or nitrogen was reduced, the grain yield of Hyou 518 would be significantly reduced. The grain yield could be maintained by appropriately reducing irrigation amount under high NFAR or maintaining continuous flooding cultivation amount under medium NFAR in Hanyou 73 (WDR). The improvement of root morphology and physiology (root dry weight, root oxidation activity, root bleeding rate, root total absorbing surface area, root active absorbing surface area, and Z + ZR contents in roots) was a considerable factor for the high yield under different irrigation regimes and NFAR in Hanyou 73. In the future, it is necessary to further study the response mechanism of root exudate components and concentrations, the interaction between different hormones in roots, and soil microorganisms of WDR under irrigation and nitrogen. We must find out the cultivation method suitable for WDR with stable yield, with the decrease of agricultural water resources, which can produce more food under the premise of saving water resources.

## Data availability statement

The original contributions presented in the study are included in the article/supplementary files, further inquiries can be directed to the corresponding author/s.

## Author contributions

DH: Conceptualization, Data curation, Formal Analysis, Investigation, Software, Writing – original draft, Writing – review & editing. KL: Conceptualization, Data curation, Formal Analysis, Investigation, Software, Writing – original draft, Writing – review & editing. SL: Methodology, Resources, Supervision, Writing – original draft. JL: Methodology, Resources, Supervision, Writing – review & editing. JT: Methodology, Resources, Supervision, Writing – review & editing. QB: Investigation, Software, Visualization, Writing – review & editing. AZ: Investigation, Software, Visualization, Writing – original draft. XY: Data curation, Investigation, Writing – review & editing. JB: Funding acquisition, Methodology, Project administration, Resources, Supervision, Writing – review & editing. LL: Funding acquisition, Methodology, Project administration, Resources, Supervision, Writing – review & editing.
